# Association of the second birth mode of delivery and interval with maternal pelvic floor changes: a prospective cohort study

**DOI:** 10.1186/s12884-024-06366-6

**Published:** 2024-03-07

**Authors:** Xiaoli Wu, Xiu Zheng, Xiaohong Yi, Bolin Fan

**Affiliations:** Department of Ultrasonography, Panzhihua Central Hospital, No.34 Yikang Street, Panzhihua, Sichuan 617067 China

**Keywords:** Pelvic floor, Pelvic floor disorders, Delivery, obstetrics, Birth intervals

## Abstract

**Background:**

This study aimed to explore the association of the second birth delivery mode and interval with maternal pelvic floor changes.

**Methods:**

This prospective cohort study included women who had a first delivery and were in weeks 36–41 of a subsequent pregnancy at Panzhihua Central Hospital between July 2017 and June 2018. The primary outcomes of the study were the hiatus area at 6 months postpartum and bladder neck (mm) at rest and during a maximum Valsalva maneuver.

**Results:**

There were 112 women with vaginal delivery and 182 with Cesarean section. The hiatus area and hiatus circumference decreased at all time points (all *P* < 0.001). The women with Cesarean section had a smaller hiatus area and circumference (*P* < 0.001 and *P* < 0.001). The hiatus diameters decreased with time in both groups (all *P* < 0.001) and were smaller after Cesarean section (both *P* < 0.001). The bladder neck at maximum Valsalva increased with time (all *P* < 0.001) without significant differences between the two groups. Finally, the proportion of patients with POP-Q stage 0/I increased with time in both groups (all *P* < 0.001), with the proportions being higher in the Cesarean group (*P* = 0.002). The birth interval was negatively correlated with the hiatus area (B=-0.17, 95%CI: -0.25, -0.08, *P* < 0.001) and positively correlated with the bladder neck at rest (B = 0.22, 95%CI: 0.08, 0.35, *P* = 0.001) and at maximum Valsalva (B = 0.85, 95%CI: 0.65, 1.05, *P* < 0.001).

**Conclusions:**

In conclusion, the mode of delivery at the second birth could influence the hiatus area and circumference and bladder neck size. The birth interval was negatively correlated with the hiatus area and positively correlated with the bladder neck at rest and at maximum Valsalva.

## Background

Pelvic floor dysfunction (PFD) affects about 50% of childbearing women [1]. Sexual dysfunction can affect 40% of reproductive-age women [2]. By age 80, 19% of women will have at least one surgical intervention for PFD or pelvic organ prolapse (POP) [3–5]. The exact pathophysiology of PFD is not completely understood. The mechanical aspects of PFD involve the widening of the levator hiatus and laxity of the pelvic floor [6]. POP is associated with increased abdominal pressure due to obesity or straining to defecate [7]. Perineal ultrasound can reveal the changes in pelvic floor structures after delivery [8, 9]. Ultrasound is inexpensive, widely available, portable, and easy to operate and could provide useful indexes for determining the risk of PFD or POP [10, 11].

The musculoskeletal changes associated with pregnancy and the direct injuries inherited from vaginal childbirth can also lead to PFD [12–14]. Indeed, women with obstetric and sphincter injuries have a high risk of PFD 1 year after delivery [15]. On the other hand, Cesarean section is associated with a lower risk of PFD compared with vaginal delivery [16–19].

The impact of a second delivery on the pelvic floor is poorly understood. Mathematical models suggest that the greatest part of the trauma causing PFD is due to the first delivery [14]. Still, epidemiological data suggest that the second and subsequent deliveries increase the risk of POP [20, 21] and PFD [22]. On the other hand, Jundt et al. [23] reported that significant changes in the pelvic floor occur 27 months on average after delivery but that subsequent deliveries do not compromise the pelvic floor further. Horak et al. [24] reported that a second delivery does not have additional major impacts on bladder support or levator function. A better understanding of the impact of a second delivery on PFD is important considering the large number of Chinese women seeking a second child after the changes in China’s birth policies [25], but the influences of the second birth delivery mode and interval on the pelvic floor are poorly understood.

Therefore, this study aimed to explore the impact of the second birth delivery mode and interval on maternal pelvic floor changes. The results could provide valuable information for the management of women having a second child.

## Methods

### Study design and participants

This prospective cohort study included women who had a first delivery and were in weeks 36–41 of a subsequent pregnancy and were undergoing prenatal checkups at Panzhihua Central Hospital (Panxi Region, China) between July 2017 and June 2018. The study was approved by the Medical Ethics Committee of Panzhihua Central Hospital. All participants signed the written informed consent form.

The inclusion criteria were 1) > 18 years of age, 2) singleton pregnancy, 3) full-term gestation (36–41 weeks), 4) having given birth once, and 5) signing the informed consent form. The exclusion criteria were (1) history of pelvic surgery or pelvic floor injury, (2) any serious disease that can have a significant impact on pelvic floor recovery (e.g., respiratory diseases and chronic cough can increase the intra-abdominal pressure), serious malnutrition, any disease leading to muscular weakness, or developmental malformation that can affect the pelvic floor muscles, or (3) any other reasons deemed unsuitable for participation by the investigators.

The participants were grouped according to the mode of delivery (vaginal vs. cesarean section). The indications for cesarean section surgery included (1) any pathological or physiological conditions where vaginal delivery is not possible or appropriate, (2) fetal distress, and (3) full-term single pregnancies and cesarean section was requested by the pregnant woman.

### Ultrasonography and POP-Q assessment

**Fig. 1:**
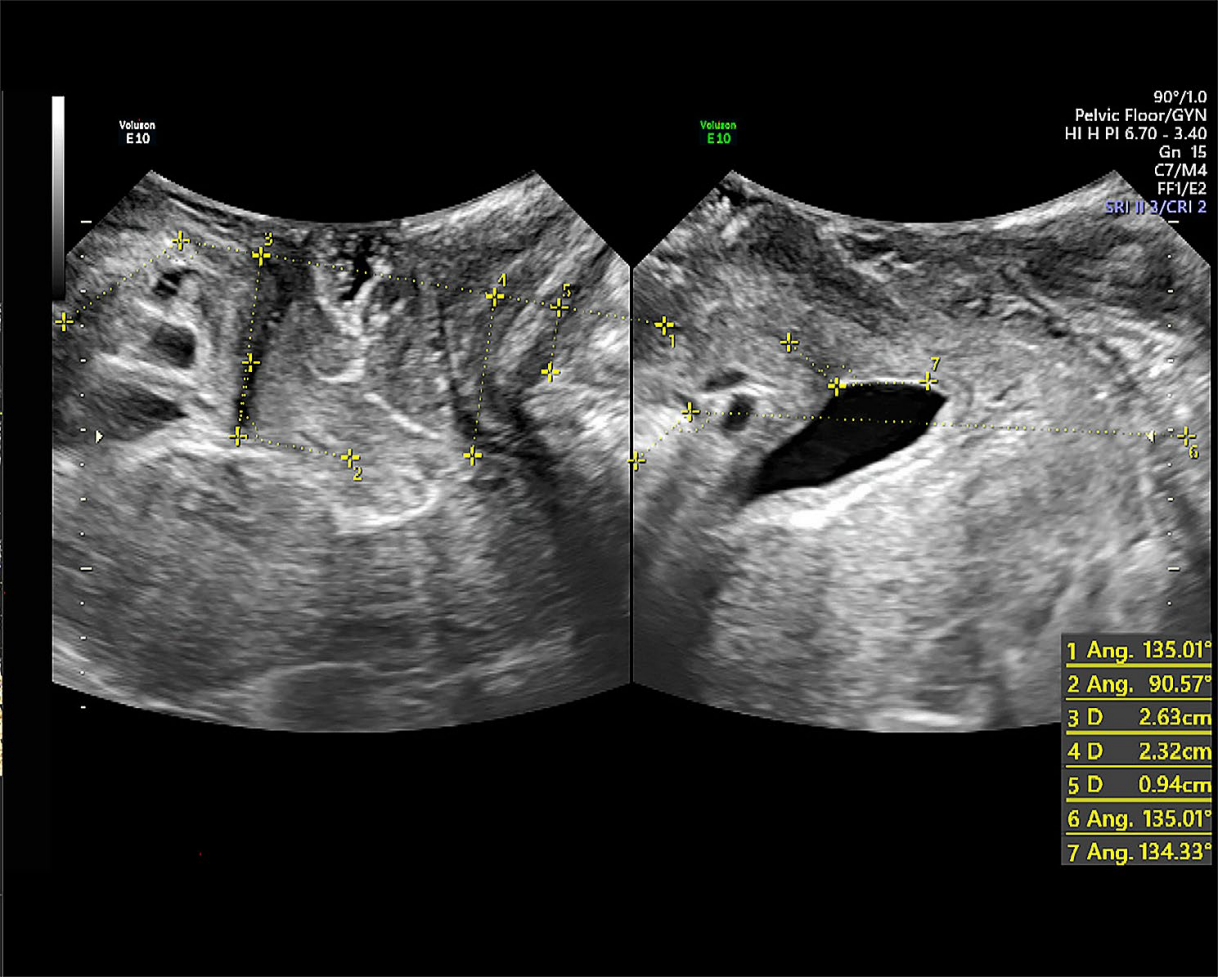
Two-dimensional ultrasound image. (Left) At rest; (Right) At maximum Valsalva. **1 **: reference line; **2 **: posterior angle of the bladder and urethra; **3 **: distance between the cervix and the reference line; **4 **: distance between the external cervix and the reference line; **5 **: distance between the ampulla of the rectum and the reference line; **6 **: reference line; **7 **: posterior angle of the bladder and urethra

**Fig. 2:**
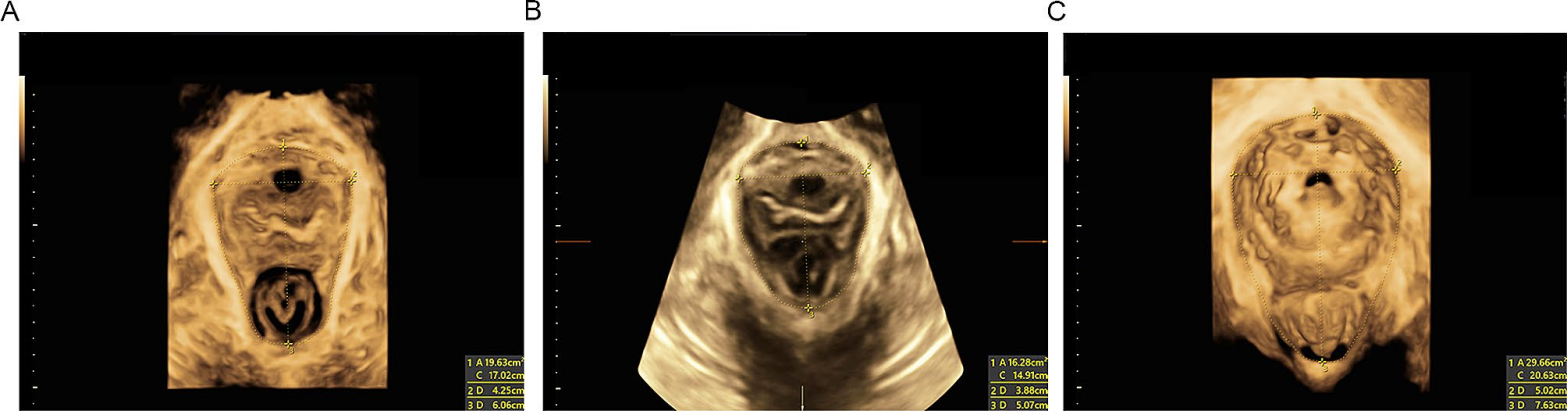
Four-dimensional hiatus sonography (1 A: basin septal hole area; 1 C: basin septal hole circumference; 2: basin septal hole diameter; 3: anterior and posterior diameters of basin septa). (**A**) Resting state. (**B**) Retracted anus state. (**C**) Maximum Valsalva state

All patients underwent routine pregnancy and prenatal ultrasound examinations at the study hospital. They were enrolled in the study at weeks 36–41. Their clinical data were obtained from their medical charts or through inquiries. The ultrasounds were performed before birth and 1, 3, and 12 months postpartum. All perineal ultrasonography examinations were performed by two experienced sonographers. Before the examination, the participant was asked to empty her bladder 30–40 min before the test to moderately fill the bladder to ensure that the urine volume in the bladder was 10–50 ml. Imaging was performed with the patients in dorsal lithotomy, with the hips flexed and slightly abducted. In the mid-sagittal plane of the pelvic floor, with the posterior inferior border of the pubic symphysis as the origin, the central axis of the pubic symphysis and the X-axis line passing through the posterior inferior border of the pubic symphysis form a 45°, and a rectangular coordinate system was established to estimate the location and activity of the bladder neck and urethra [26]. The posterior angle of the bladder, the distance from the bladder neck to the reference line, and the distance from the cervix to the reference line were measured along with calculation of the urethral rotation angle, bladder neck mobility, etc. (Fig. 1) to observe whether there was infundibulum, cystocele, and uterine prolapse and the degree of the internal urethral orifice. Then, four-dimensional ultrasound imaging was performed to freeze the images in the patient’s resting state, anal retraction state, and maximum Valsalva state, respectively, and adjust the region of interest to include the minimum gap plane between the posterior side of the pubic symphysis and the anterior side of the anorectal angle, respectively. The area, anterior-posterior diameter, and left-right diameter of the pelvic diaphragmatic hiatus were measured (Fig. 2). Finally, the TUI-VCI imaging mode was used to observe the levator hiatus and the continuity of the levator ani. The ultrasound pictures after the examination were all consulted by the same group of ultrasound specialists who had been engaged in the specialty for more than 3 years, and the opinions were unified by consulting or asking other professional doctors.

All patients were scored by the same gynecologist at the pelvic floor rehabilitation center for POP-Q scores [27–31]. The hymen was selected as a reference (0 points), and the 6 points on the anterior wall, posterior wall, and top of the vagina were taken as the indicator points (two points Aa, Ba on the anterior wall, two points Ap, Bp on the posterior wall, and two points C, D on the top). The change in the position of the 6 o’clock position relative to the hymen was defined as a scale (indicating points located medial to the hymen margin were scored as negative numbers and those located lateral to the hymen margin were scored as positive numbers) to quantify prolapse. The total vaginal length (tvl), the height of the genital hiatus (gh), and the length of the perineal body (pb) were recorded. POP-Q stage 0: No prolapse, with Aa, Ap, Ba, and Bp all located at or above − 3 cm, and point C or D positioned between -TVL and -(TVL-2) cm. POP-Q stage 1: Prolapse is present, extending beyond stage 0. The furthest point of prolapse is within the vaginal hymen, with a distance from the hymen > 1 cm.

### Outcomes

The primary outcomes of the study were the hiatus area at 6 months postpartum and bladder neck (mm) at rest and during a maximum Valsalva maneuver. The secondary outcomes of the study were the hiatus area, anteroposterior, left-right, and girth at 1, 3, and 12 months postpartum, bladder neck (mm) at rest and maximum Valsalva maneuver, and the POP-Q scores at 1, 3, 6, and 12 months postpartum.

### Follow-up

All pregnant women underwent outpatient follow-ups at 1, 3, 6, and 12 months postpartum (these time points were used to obtain a dynamic evaluation of pelvic floor evolution over time), and 2- and 4-dimensional ultrasonography was performed at each visit to assess their pelvic function.

### Data collection

The baseline characteristics of the pregnant women were collected, including data such as age, the number of births, first-birth weight, body mass index (BMI), and abnormal symptoms. At the same time, the related data of ultrasound measurement and POP-Q score of pregnant women were collected at 1, 3, 6, and 12 months postpartum.

### Statistical analysis

Data were analyzed using Stata 17.0 (Stata Corporation, College Station, TX, USA). The continuous data were tested for normal distribution using the Kolmogorov-Smirnov method. Non-normally distributed measurement data were expressed as median (25th percentile, 75th percentile) and were tested using the Mann-Whitney U-test. Normally distributed continuous data were expressed as means ± standard deviation and were analyzed using the t-test. Categorical data were expressed as n (%) and were tested using the chi-square test or Fisher’s exact probability method. Repeated measures were analyzed using the generalized estimating equation (GEE). Linear regression was used to explore the effect of the second birth interval on the primary outcomes, adjusting for maternal age, pre-pregnancy BMI, and second birth weight. Two-sided P-values < 0.05 were considered statistically significant.

## Results

The women were grouped according to the mode of delivery: 112 with vaginal delivery and 182 with Cesarean section. Compared with the vaginal delivery group, the women in the cesarean section group were older (32.25 ± 5.37 vs. 30.42 ± 5.86 years old, *P* = 0.007) (Table 1). Most pregnant women had a history of miscarriage since they had a mean of 3.16 ± 1.19 pregnancies and were enrolled during their second full-term pregnancy. The participants had no pelvic muscle training after delivery.

**Table 1:** Characteristics of the participants

	Total (*n* = 294)	Vaginal delivery (*n* = 112)	Cesarean section (*n* = 182)	P
Age (years)	31.55 ± 5.62	30.42 ± 5.86	32.25 ± 5.37	0.007
Mean number of pregnancies	3.16 ± 1.19	3.00 ± 1.12	3.26 ± 1.22	0.064
First birth weight (kg)	3.22 ± 0.39	3.19 ± 0.37	3.25 ± 0.39	0.526
Second birth weight (kg)	3.28 ± 0.35	3.26 ± 0.31	3.29 ± 0.37	0.410
Pre pregnancy BMI	23.61 ± 2.89	23.42 ± 3.12	23.72 ± 2.75	0.388
Abnormal symptoms	131 (44.56)	57 (50.89)	74 (40.66)	0.086

BMI: body mass index

In all women, the hiatus area and hiatus circumference decreased at all time points (all *P* < 0.001). The women with Cesarean section had a smaller hiatus area and circumference (*P* < 0.001 and *P* < 0.001). The hiatus diameters decreased with time in both groups (all *P* < 0.001) and were smaller after Cesarean section (both *P* < 0.001). The bladder neck at rest was smaller at 6 and 12 months compared with 1 month (both *P* = 0.05), without significant differences between the two groups. The bladder neck at maximum Valsalva increased with time (all *P* < 0.001) without significant differences between the two groups. Finally, the proportion of patients with POP-Q stage 0/I increased with time in both groups (all *P* < 0.001), with the proportions being higher in the Cesarean group (*P* = 0.002) (Table 2).

**Table 2:** Comparison of postpartum pelvic function and POP-Q indexes in the overall populations

Parameters	Follow-up (months)	Vaginal delivery (*n* = 112)	Cesarean section (*n* = 182)	P_group_	P_time_
Hiatus area (cm^2^)	1	22.04 ± 4.16	19.02 ± 3.76	< 0.001	< 0.001
	3	19.16 ± 3.74	16.46 ± 3.33		< 0.001
	6	16.74 ± 3.18	14.54 ± 3.21		< 0.001
	12	14.35 ± 2.66	12.94 ± 3.37		< 0.001
Hiatus circumference (mm)	1	186.15 ± 21.23	173.71 ± 25.50	< 0.001	< 0.001
	3	170.15 ± 19.51	156.08 ± 26.60		< 0.001
	6	155.67 ± 33.78	145.43 ± 33.78		< 0.001
	12	139.87 ± 18.39	130.53 ± 34.67		< 0.001
Hiatus left and right diameter (cm)	1	5.35 ± 0.81	5.13 ± 0.84	< 0.001	< 0.001
	3	4.84 ± 0.74	4.61 ± 0.74		< 0.001
	6	4.47 ± 0.65	4.25 ± 0.67		< 0.001
	12	4.07 ± 0.54	3.89 ± 0.55		< 0.001
Hiatus front and rear diameter (cm)	1	6.43 ± 1.77	5.95 ± 0.69	< 0.001	< 0.001
	3	5.73 ± 0.64	5.39 ± 0.64		< 0.001
	6	5.30 ± 0.65	4.98 ± 0.67		< 0.001
	12	4.80 ± 0.59	4.53 ± 0.59		< 0.001
Bladder neck (mm) (rest)	1	17.54 ± 6.95	19.18 ± 6.25	0.073	Ref.
	3	16.78 ± 6.59	18.34 ± 6.60		0.081
	6	16.87 ± 7.22	18.08 ± 7.40		0.043
	12	17.11 ± 6.66	16.84 ± 7.03		< 0.001
Bladder neck (mm) (Fall’s)	1	-12.58 ± 11.60	-11.31 ± 9.11	0.129	Ref.
	3	-5.61 ± 10.40	-3.41 ± 10.15		< 0.001
	6	-0.40 ± 8.96	0.34 ± 7.89		< 0.001
	12	2.28 ± 6.65	2.99 ± 5.98		< 0.001
POP-Q staging (Phase 0/I)	1	48 (42.86)	92 (50.55)	0.002	0.012
	3	73 (65.18)	144 (79.12)		< 0.001
	6	101 (90.18)	173 (95.05)		< 0.001
	12	107 (95.54)	181 (99.45)		< 0.001

POP-Q: pelvic organ prolapse quantification

P_Time_: P-values for the comparisons of the data among time points between two groups

P_Group_: P-values for the comparisons between groups

The multivariable analyses showed that the birth interval was negatively correlated with the hiatus area (B=-0.17, 95%CI: -0.25, -0.08, *P* < 0.001) and positively correlated with bladder neck at rest (B = 0.22, 95%CI: 0.08, 0.35, *P* = 0.001) and at maximum Valsalva (B = 0.85, 95%CI: 0.65, 1.05, *P* < 0.001) (Table 3). After vaginal delivery, the birth interval was negatively correlated with the hiatus area (B=-0.24, 95%CI: -0.39, -0.09, *P* = 0.002) and positively correlated with bladder neck at rest (B = 0.28, 95%CI: 0.05, 0.51, *P* = 0.018) and at maximum Valsalva (B = 1.04, 95%CI: 0.67, 1.40, *P* < 0.001) (Table 3). After Cesarean section, the birth interval was positively correlated with bladder neck at maximum Valsalva (B = 0.74, 95%CI: 0.50, 0.97, *P* < 0.001) (Table 3).

**Table 3:** Effect of interval time between second births on pelvic function

Pelvic function indicators	Model ^a^	B (95% CI) ^#^	P
Hiatus area (cm^2^)	All	-0.17 (-0.25, -0.08)	< 0.001
	Vaginal delivery	-0.24 (-0.39, -0.09)	0.002
	Cesarean section	-0.07 (-0.17 0.03)	0.148
Bladder neck (mm) (rest)	All	0.22 (0.08, 0.35)	0.001
	Vaginal delivery	0.28 (0.05, 0.51)	0.018
	Cesarean section	0.17 (-0.0004, 0.34)	0.051
Bladder neck (mm) (Fall’s)	All	0.85 (0.65, 1.05)	< 0.001
	Vaginal delivery	1.04 (0.67, 1.40)	< 0.001
	Cesarean section	0.74 (0.50, 0.97)	< 0.001

a: indicates that variables such as age, second birth weight and pre pregnancy BMI were adjusted

## Discussion

The study showed that the mode of delivery at the second birth could influence the hiatus area and circumference and bladder neck size. The birth interval was negatively correlated with the hiatus area and positively correlated with the bladder neck at rest and at maximum Valsalva.

Romano et al. [32] advocate that there are three postpartum stages. The first stage is 6–12 h after delivery and includes acute events such as hemorrhage, uterine inversion, amniotic fluid embolism, and eclampsia. The second stage lasts 2–6 weeks and involves the recovery of hemodynamics, genitourinary structures, metabolism, and emotions. The third phase would last up to 6 months, in which recovery changes are gradual. In fact, the present study suggests that the recovery process could even be longer and take years. Indeed, in the present study, the women with Cesarean section showed significantly fewer detrimental consequences after a second delivery, as indicated by better POP-Q stages, suggesting that their pelvic floor was less damaged. It is supported by two meta-analyses that showed that vaginal delivery was directly related to pelvic floor disorders [19, 33], but the present study suggests that it remains true even after a second delivery.

Blomquist et al. [34] reported that the women undergoing Cesarean delivery were at a significantly lower risk of PFD and POP than those with spontaneous vaginal delivery. At 6 years after delivery, vaginal delivery is associated with urinary incontinence, while a Cesarean section is associated with sexual and urination pain [35]. Zhao et al. [36] showed that vaginal delivery was an independent risk factor for pelvic floor muscle injury. In the present study, differences were observed between vaginal and Cesarean deliveries in hiatus dimensions, bladder neck, and POP-Q scores, with general trends toward less damage after Cesarean and more women with POP-Q stage 0/I compared with vaginal delivery. It is supported by Chan et al. [37]. The exact symptoms of PFD were not assessed in the present study. Future studies will have to examine the symptoms in relation to the interval length between deliveries.

Although data in the literature are rare regarding the impact of the delivery interval on the pelvic floor, studies indicated that the risk of uterine rupture decreased with the delivery interval, with rates of 4.8% at ≤ 12 months, 2.7% at 13–24 months, and 0.9% at ≥ 25 months [38], indicating healing of the structures with time beyond the first 6 months after delivery. The present study indicated a negative correlation between the interval and the hiatus area and positive correlations with bladder neck at rest and at maximum Valsalva, suggesting less detrimental effects of delivery with longer intervals, probably because of healing of the pelvic structures and functions. These correlations were observed with vaginal delivery, but only with maximum Valsalva in patients with Cesarean section, probably because of the smaller damage to the pelvic function. Still, additional studies are necessary to examine this issue.

This study has some limitations. Although it was a prospective cohort study, the women were from a single center, and the sample size was relatively small. Women with any aggravating factor were excluded, decreasing the generalizability of the results. Although all measurements were performed by the same sonographers, ultrasound is operator-dependent, and the position of the patients can influence the measurements. A symptom assessment was not performed, and the presence of urinary incontinence during pregnancy, variations in weight during pregnancy, and the mode of delivery at the first pregnancy were not collected. The first follow-up was at 1 month, which was in the 42-day puerperal period, and it is obvious that the tissues had not yet returned to their original place. Finally, factors like episiotomy and pelvic floor muscle training were not evaluated. The sample size was relatively small and did not allow reliable subgroup analyses. Additional studies are necessary to evaluate these factors.

In conclusion, the mode of delivery at the second birth could influence the hiatus area and circumference and bladder neck size. The birth interval was negatively correlated with the hiatus area and positively correlated with the bladder neck at rest and at maximum Valsalva. These correlations were also observed for vaginal delivery, but only the birth interval was only positively correlated with bladder neck at maximum Valsalva after Cesarean section.

## Data Availability

All data generated or analyzed during this study are included in this published article.

## Abbreviations

PFD: Pelvic Floor Dysfunction

## References

[1] Hagen S, Stark D. Conservative prevention and management of pelvic organ prolapse in women. Cochrane Database Syst Rev 2011:CD003882. 10.1002/14651858.CD003882.pub4 PMC12621084 22161382

[2] McCool-Myers M, Theurich M, Zuelke A, Knuettel H, Apfelbacher C (2018). Predictors of female sexual dysfunction: a systematic review and qualitative analysis through gender inequality paradigms. BMC Womens Health.

[3] Fialkow MF, Newton KM, Lentz GM, Weiss NS (2008). Lifetime risk of surgical management for pelvic organ prolapse or urinary incontinence. Int Urogynecol J Pelvic Floor Dysfunct.

[4] Olsen AL, Smith VJ, Bergstrom JO, Colling JC, Clark AL (1997). Epidemiology of surgically managed pelvic organ prolapse and urinary incontinence. Obstet Gynecol.

[5] Smith FJ, Holman CD, Moorin RE, Tsokos N (2010). Lifetime risk of undergoing surgery for pelvic organ prolapse. Obstet Gynecol.

[6] Boyadzhyan L, Raman SS, Raz S (2008). Role of static and dynamic MR imaging in surgical pelvic floor dysfunction. Radiographics.

[7] Spence-Jones C, Kamm MA, Henry MM, Hudson CN (1994). Bowel dysfunction: a pathogenic factor in uterovaginal prolapse and urinary stress incontinence. Br J Obstet Gynaecol.

[8] Asfour V, Digesu GA, Fernando R, Khullar V (2020). Ultrasound imaging of the perineal body: a useful clinical tool. Int Urogynecol J.

[9] Fonti Y, Giordano R, Cacciatore A, Romano M, La Rosa B (2009). Post partum pelvic floor changes. J Prenat Med.

[10] Hainsworth AJ, Solanki D, Schizas AM, Williams AB (2015). Total pelvic floor ultrasound for pelvic floor defaecatory dysfunction: a pictorial review. Br J Radiol.

[11] Dalpiaz O, Curti P (2006). Role of perineal ultrasound in the evaluation of urinary stress incontinence and pelvic organ prolapse: a systematic review. Neurourol Urodyn.

[12] Lal M, C HM, Callender R, Radley S (2003). Does cesarean delivery prevent anal incontinence?. Obstet Gynecol.

[13] Yilmaz E, Nas T, Korucuoglu U, Guler I (2008). Manometric evaluation of anal sphincter function after vaginal and cesarean delivery. Int J Gynaecol Obstet.

[14] Dietz HP, Gillespie AV, Phadke P (2007). Avulsion of the pubovisceral muscle associated with large vaginal tear after normal vaginal delivery at term. Aust N Z J Obstet Gynaecol.

[15] Huber M, Malers E, Tunon K (2021). Pelvic floor dysfunction one year after first childbirth in relation to perineal tear severity. Sci Rep.

[16] Memon H, Handa VL (2012). Pelvic floor disorders following vaginal or cesarean delivery. Curr Opin Obstet Gynecol.

[17] Handa VL, Blomquist JL, Knoepp LR, Hoskey KA, McDermott KC, Munoz A (2011). Pelvic floor disorders 5–10 years after vaginal or cesarean childbirth. Obstet Gynecol.

[18] Handa VL, Blomquist JL, McDermott KC, Friedman S, Munoz A (2012). Pelvic floor disorders after vaginal birth: effect of episiotomy, perineal laceration, and operative birth. Obstet Gynecol.

[19] Barca JA, Bravo C, Pintado-Recarte MP, Asunsolo A, Cueto-Hernandez I, Ruiz-Labarta J, Bujan J, Ortega MA, De Leon-Luis JA. Pelvic floor morbidity following vaginal delivery versus cesarean delivery: systematic review and Meta-analysis. J Clin Med 2021, 10. 10.3390/jcm10081652 PMC8070303 33924472

[20] Patel DA, Xu X, Thomason AD, Ransom SB, Ivy JS, DeLancey JO (2006). Childbirth and pelvic floor dysfunction: an epidemiologic approach to the assessment of prevention opportunities at delivery. Am J Obstet Gynecol.

[21] Mant J, Painter R, Vessey M (1997). Epidemiology of genital prolapse: observations from the Oxford Family Planning Association Study. Br J Obstet Gynaecol.

[22] Rortveit G, Daltveit AK, Hannestad YS, Hunskaar S, Norwegian ES (2003). Urinary incontinence after vaginal delivery or cesarean section. N Engl J Med.

[23] Jundt K, Scheer I, von Bodungen V, Krumbachner F, Friese K, Peschers UM (2010). What harm does a second delivery to the pelvic floor?. Eur J Med Res.

[24] Horak TA, Guzman-Rojas RA, Shek KL, Dietz HP (2014). Pelvic floor trauma: does the second baby matter?. Ultrasound Obstet Gynecol.

[25] Zeng Y, Hesketh T (2016). The effects of China’s universal two-child policy. Lancet.

[26] Dietz HP, Hoyte LPJ, Steensma AB (2008). Atlas of Pelvic Floor Ultrasound.

[27] Maher C, Feiner B, Baessler K, Schmid C. Surgical management of pelvic organ prolapse in women. Cochrane Database Syst Rev 2013:CD004014. 10.1002/14651858.CD004014.pub5 23633316

[28] Muir TW, Stepp KJ, Barber MD (2003). Adoption of the pelvic organ prolapse quantification system in peer-reviewed literature. Am J Obstet Gynecol.

[29] Treszezamsky AD, Rascoff L, Shahryarinejad A, Vardy MD (2010). Use of pelvic organ prolapse staging systems in published articles of selected specialized journals. Int Urogynecol J.

[30] Vierhout ME, Stoutjesdijk J, Spruijt J (2006). A comparison of preoperative and intraoperative evaluation of patients undergoing pelvic reconstructive surgery for pelvic organ prolapse using the pelvic organ prolapse quantification system. Int Urogynecol J Pelvic Floor Dysfunct.

[31] Manonai J, Mouritsen L, Palma P, Contreras-Ortiz O, Korte JE, Swift S (2011). The inter-system association between the simplified pelvic organ prolapse quantification system (S-POP) and the standard pelvic organ prolapse quantification system (POPQ) in describing pelvic organ prolapse. Int Urogynecol J.

[32] Romano M, Cacciatore A, Giordano R, La Rosa B (2010). Postpartum period: three distinct but continuous phases. J Prenat Med.

[33] Yang XJ, Sun Y (2019). Comparison of caesarean section and vaginal delivery for pelvic floor function of parturients: a meta-analysis. Eur J Obstet Gynecol Reprod Biol.

[34] Blomquist JL, Munoz A, Carroll M, Handa VL (2018). Association of Delivery Mode with Pelvic Floor disorders after Childbirth. JAMA.

[35] Baud D, Sichitiu J, Lombardi V, De Rham M, Meyer S, Vial Y, Achtari C (2020). Comparison of pelvic floor dysfunction 6 years after uncomplicated vaginal versus elective cesarean deliveries: a cross-sectional study. Sci Rep.

[36] Zhao Y, Zou L, Xiao M, Tang W, Niu HY, Qiao FY (2018). Effect of different delivery modes on the short-term strength of the pelvic floor muscle in Chinese primipara. BMC Pregnancy Childbirth.

[37] Chan SSC, Cheung RYK, Lee LL, Chung TKH (2018). Longitudinal pelvic floor biometry: which factors affect it?. Ultrasound Obstet Gynecol.

[38] Bujold E, Mehta SH, Bujold C, Gauthier RJ (2002). Interdelivery interval and uterine rupture. Am J Obstet Gynecol.

